# Tryptophan end-tagging for promoted lipopolysaccharide interactions and anti-inflammatory effects

**DOI:** 10.1038/s41598-017-00188-7

**Published:** 2017-03-16

**Authors:** Shalini Singh, Aritreyee Datta, Artur Schmidtchen, Anirban Bhunia, Martin Malmsten

**Affiliations:** 1 Department of Pharmacy, Uppsala University, SE-75232 Uppsala, Sweden; 2 Department of Biophysics, Bose Institute, P-1/12 CIT Scheme VII (M), Kolkata, 700054 India; 3 Division of Dermatology and Venereology, Department of Clinical Sciences, Lund University, SE-221 84, Lund, Sweden; 4 Lee Kong Chian School of Medicine, Nanyang Technological University, 11, Mandalay Road, 308232 Singapore

## Abstract

The objective of the present study is the investigation of possibilities for boosting peptide anti-inflammatory effects by tryptophan end-tagging, including identification of underlying mechanisms for this. In doing so, effects of tryptophan end-tagging of KYE21 (KYEITTIHNLFRKLTHRLFRR), a peptide derived from heparin co-factor II, on membrane and lipopolysaccharide (LPS) interactions were investigated by ellipsometry, NMR, fluorescence spectroscopy, and circular dichroism measurements. Through its N-terminal W stretch, WWWKYE21 displays higher membrane binding, liposome rupture, and bacterial killing than unmodified KYE21. Analogously, W-tagging promotes binding to *E. coli* LPS and to its endotoxic lipid A moiety. Furthermore, WWWKYE21 causes more stable peptide/LPS complexes than KYE21, as evidenced by detailed NMR studies, adopting a pronounced helical conformation, with a large hydrophobic surface at the N-terminus due to the presence of W-residues, and a flexible C-terminus due to presence of several positively charged arginine residues. Mirroring its increased affinity for LPS and lipid A, WWWKYE21 displays strongly increased anti-inflammatory effect due to a combination of direct lipid A binding, peptide-induced charge reversal of cell membranes for LPS scavenging, and peptide-induced fragmentation of LPS aggregates for improved phagocytosis. Importantly, potent anti-inflammatory effects were observed at low cell toxicity, demonstrated for both monocytes and erythrocytes.

## Introduction

Host defense peptides constitute a key part of the innate immune system^1, 2^, displaying fast and broad-spectrum antimicrobial effects through direct membrane lysis, as well as anti-inflammatory and other effects^3–5^. While the antimicrobial effects of such peptides are becoming increasingly understood, the mechanisms underlying anti-inflammatory properties of host defense peptides remain unclear. Due to inflammatory properties of lipopolysaccharide (LPS) in Gram-negative bacteria, lipoteichoic acid (LTA) in Gram-positive bacteria, and zymosan in fungi^6, 7^, various approaches have been undertaken to investigate peptide interactions with these inflammatory (lipo)polysaccharides. From such studies, it seems clear that anti-inflammatory peptides must display substantial binding to the pro-inflammatory compounds. Having said that, it is also becoming increasingly clear that such binding is only a necessary, but not a sufficient, criterion for anti-inflammatory effects of host defense peptides, and that additional processes, such as peptide-induced membrane scavenging of LPS and peptide-facilitated phagocytosis of LPS, may also play important roles^8–10^.

Of the pro-inflammatory (lipo)polysaccharides, LPS, a major component of the outer leaflet of Gram-negative bacteria, is the most investigated^11^. LPS is negatively charged through its carboxyl and phosphate groups and contains a hydrophobic lipid moiety (lipid A), anchoring it to the outer membrane^11^. LPS triggers inflammation through binding to LPS-binding protein (LBP), which is subsequently recognized by CD14 at the cell surface of monocytes/macrophages and interacting with the Toll-like receptors/myeloid differentiation protein-2 (TLR4/MD2) complex, inducing up-regulation of NF-κB and pro-inflammatory cytokines^12, 13^. In addition to this classical activation pathway, however, also LPS aggregation has been found to play an important role in inflammation triggering^14^. As the poor aqueous solubility of hydrophobic lipid A moieties drives such LPS aggregation, these are expected to localize primarily in the core of the thread-like LPS aggregates. Furthermore, it has been demonstrated that the LBP has a binding site for lipid A^15^. Therefore, if lipid A is localized in the core of the LPS aggregates, LBP binding to lipid A is precluded due to steric interactions originating from the LPS polysaccharide chains protruding from the LPS aggregates. Thus, other mechanisms must be involved as well to explain the increased inflammatory effects caused by LPS aggregates, as also discussed previously in ref. 14.

In an effort to further elucidate mechanisms underlying anti-inflammatory properties of host defense peptides, we previously identified KYE28 (KYEITTIHNLFRKLTHRLFRRNFGYTLR), derived from heparin cofactor II (HCII), as a peptide displaying potent antimicrobial and anti-inflammatory effects^16, 17^. In a biophysical investigation on the mechanisms underlying this, KYE28 was compared with two truncated peptide variants, i.e., KYE21 (KYEITTIHNLFRKLTHRLFRR), displaying partially retained antimicrobial and anti-inflammatory effects, and NLF20 (NLFRKLTHRLFRRNFGYTLR), displaying retained antimicrobial but only a fraction of the anti-inflammatory effect^9^. Thus, the anti-inflammatory properties of KYE28 were primarily localized in its N-terminus^9, 16, 17^. From investigations of peptide binding to phospholipid membranes, LPS, and lipid A, as well as effects on membrane charge and LPS aggregation, three mechanisms for the anti-inflammatory properties of KYE28 was inferred, i.e., (i) direct binding to LPS and lipid A, (ii) peptide-induced charge reversal of membrane surfaces, and (iii) fragmentation and densification of LPS aggregates, presumably through preventing LBP binding and providing alternatives to the NF-κB-generating pathway, respectively.

Considering this, as well as our previous findings that W-tagging provides a way to dramatically increase antimicrobial properties of peptides^18^, we speculated that such modifications could be used to enhance the *anti-inflammatory* properties of KYE21. In order to clarify the effect of W-tagging, KYE21 was compared to WWWKYE21 (WWWKYEITTIHNLFRKLTHRLFRR), using a battery of physicochemical methodologies, including ellipsometry, NMR, circular dichroism (CD), and fluorescence spectroscopy, comparing the results thus obtained with results on antimicrobial effects, anti-inflammatory effects, and cell toxicity.

## Results

### Membrane binding, destabilization, and antimicrobial effects

Based on previous findings of the anti-inflammatory properties of the peptide KYE28 being located primarily at its N-terminus^9^, as well as of dramatically improved antimicrobial effects at retained low toxicity against human cells after W-tagging^18, 19^, we hypothesized that W-tagging may be used also for boosting also the anti-inflammatory properties of KYE21. First, however, effects of W-tagging on membrane interactions of KYE21 were investigated for baseline.

**Figure 1:**
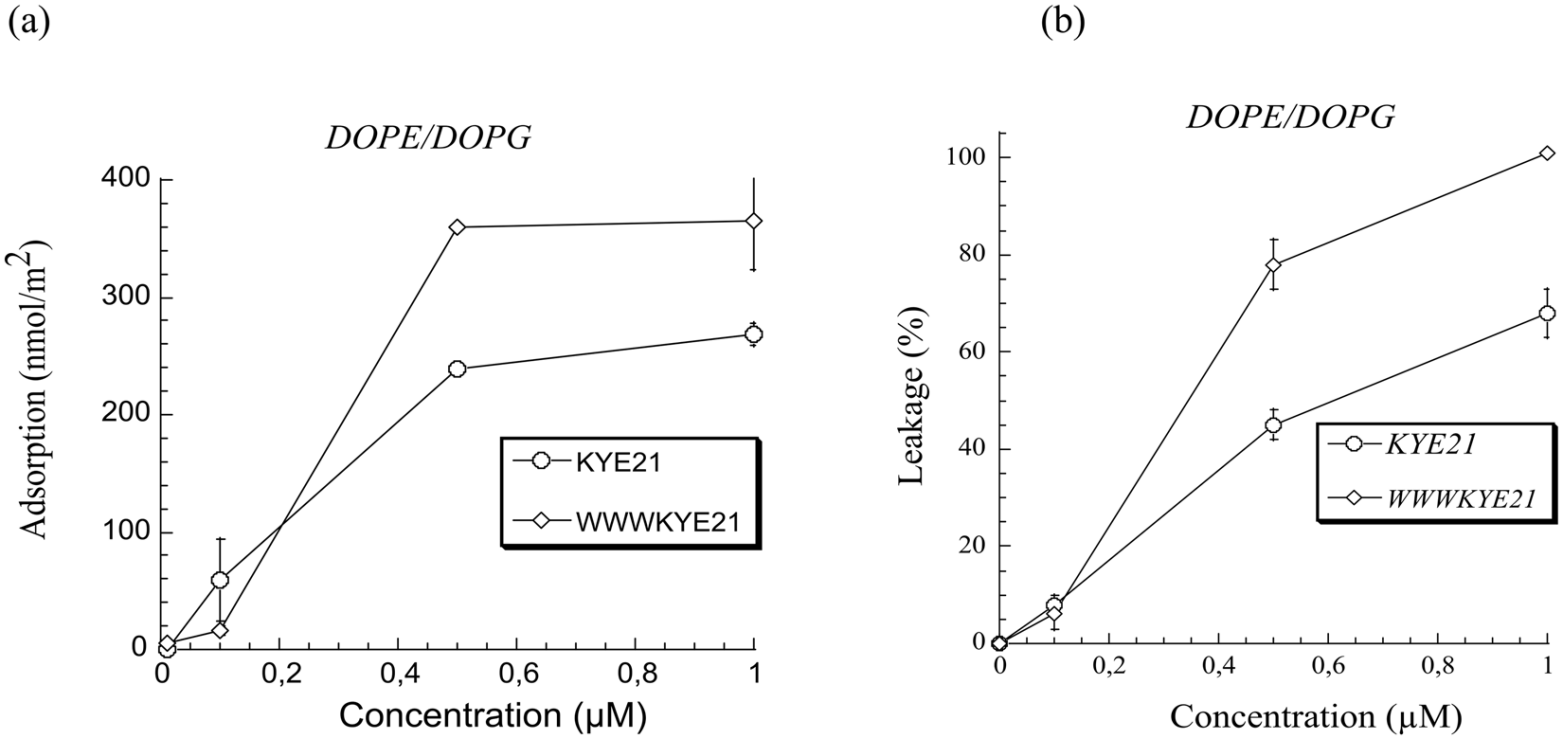
(**a**) Ellipsometry results on peptide binding to supported DOPE/DOPG (75/25 mol/mol) lipid bilayers in 10 mM Tris, pH 7.4. (**b**) Peptide-induced leakage of anionic DOPE/DOPG (75/25 mol/mol) liposomes in 10 mM Tris, pH 7.4. (n ≥ 2; For data points where error bars are not seen, these are smaller than the legend size).

**Figure 2:**
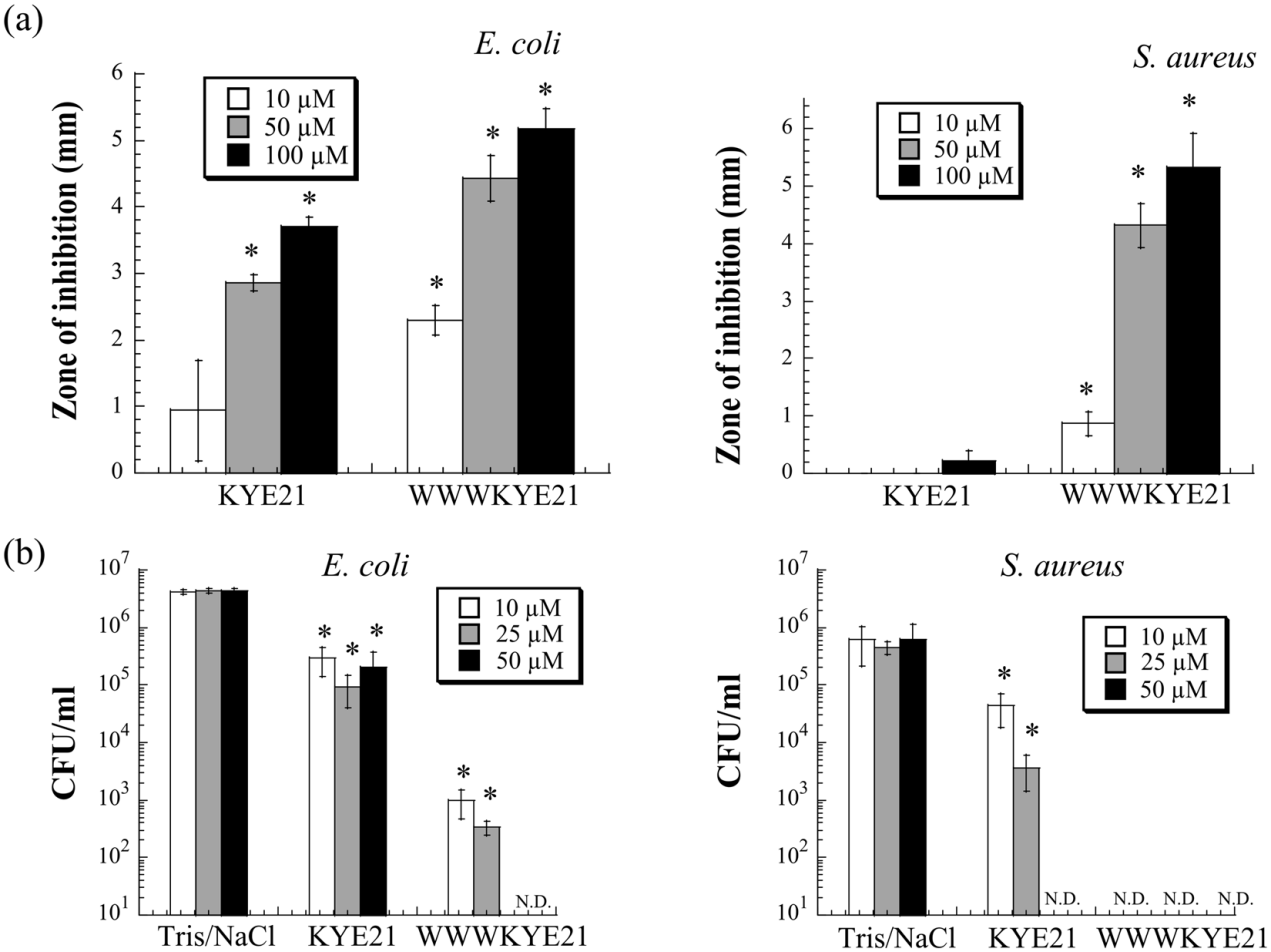
Antimicrobial activity against *E. coli* ATCC 25922 (left) and *S. aureus* ATCC 29213 (right), as determined by radial diffusion assay (RDA; **a**) and viable count assay (VCA; **b**) in 10 mM Tris, pH 7.4, 150 mM NaCl. “N.D.”: Non-detectable. (n = 3; mean ± SD shown) *p < 0.05 compared to absence of peptide. Note the logaritmic scale in **b**).

As demonstrated by ellipsometry measurements, peptide binding to DOPE/DOPG bilayers was substantially higher for WWWKYE21 than for KYE21 (Fig. 1a), reaching saturation binding at ≈360 and 270 nmol/m^2^, respectively, corresponding to peptide:lipid ratios of 1:18 and 1:13, respectively. As a result of the increased peptide binding density, liposome leakage is higher for WWWKYE21 (Fig. 1b). Analogously, WWWKYE21 displayed enhanced antimicrobial effects (caused by peptide-induced membrane lysis^19^) against Gram-negative *E. coli* and Gram-positive *S. aureus*, as demonstrated in both radial diffusion assay (Fig. 2a) and matrix-free viable count assays (Fig. 2b). Although this was observed for both Gram-negative *E. coli* and Gram-positive *S. aureus*, both RDA and VCA indicate that the antimicrobial boosting effect caused by the W-tagging is more pronounced for the latter bacteria. Quantitatively, WWWKYE21 is potently membrane-disruptive and antimicrobial, considering the high ionic strength conditions used for these experiments, illustrating the importance of non-electrostatic driving forces for membrane binding and destabilization.

### Peptide binding to LPS and lipid A

**Figure 3:**
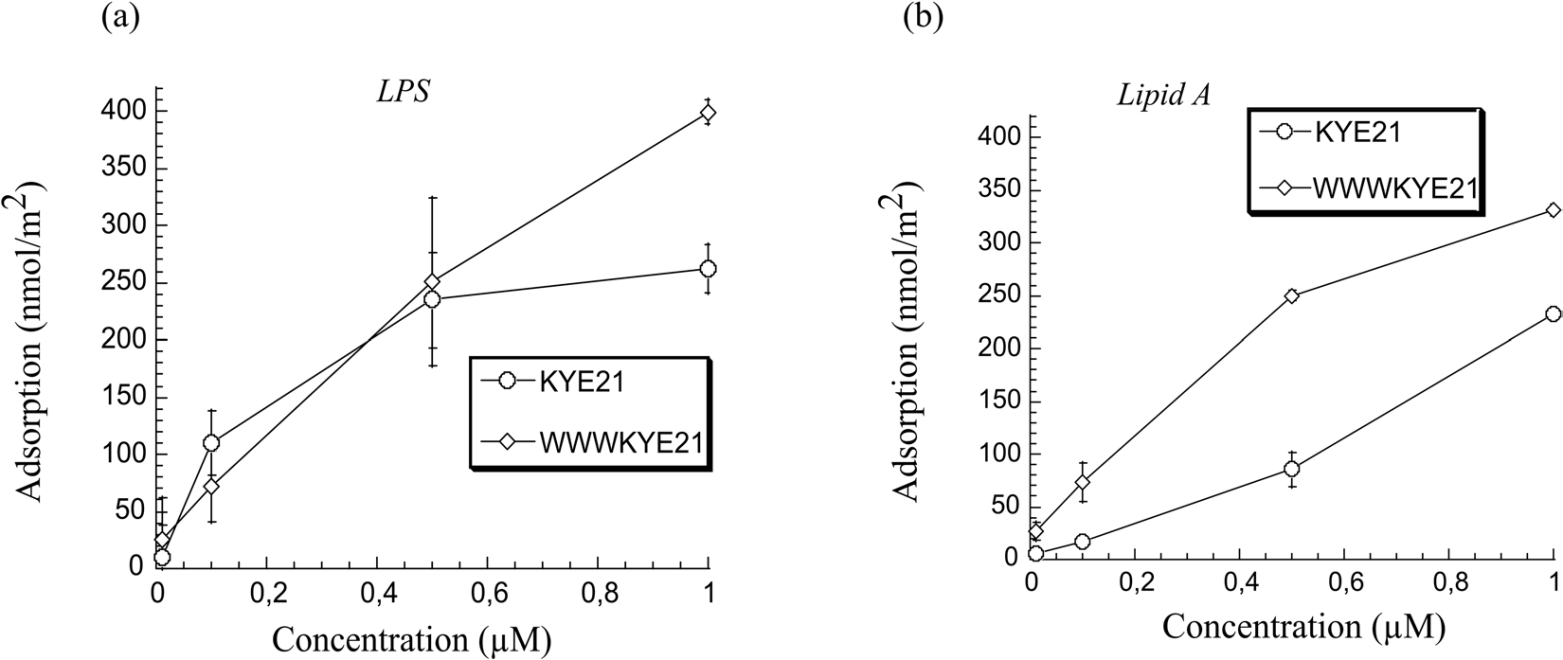
Peptide binding to LPS (**a**) and lipid A (**b**) in 10 mM Tris, pH 7.4. (n ≥ 2).

In analogy to bacteria-mimicking anionic DOPE/DOPG bilayers (z ≈ −40 mV), both KYE21 and WWWKYE21 bind to *E. coli* LPS (Fig. 3a) and its endotoxic lipid A moiety (Fig. 3b). For the polysaccharide region of LPS, peptide binding is due to negatively charged phosphate and carboxyl groups, while for lipid A, binding is due to both phosphate groups and the hydrophobic nature of lipid A^10^. Notably, a pronounced binding promotion due to W-tagging is observed for lipid A, indicating the importance of hydrophobic interactions.

### Peptide structure

**Figure 4:**
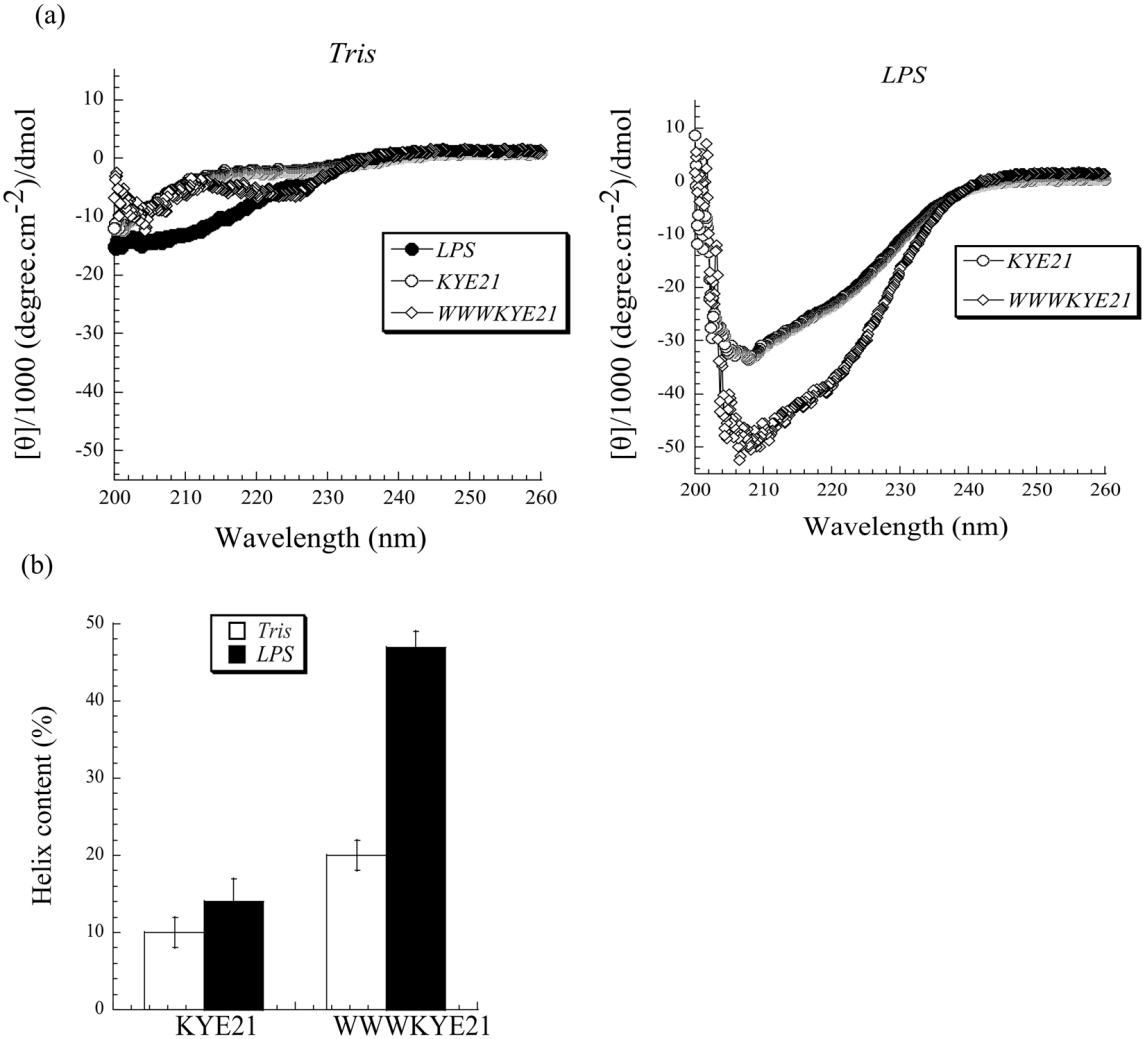
(**a**) CD spectra in 10 mM Tris, pH 7.4, with and without *E. coli* LPS. Shown also are CD spectra for LPS in 10 mM Tris, pH 7.4. (**b**) Helix content for the indicated peptides in buffer and in the presence of *E. coli* LPS (0.2 mg/ml). (n ≥ 2).

CD experiments demonstrate that the peptides investigated are largely disordered in buffer, with low (<15%) helix content. On binding to LPS, however, induction of ordered (largely α-helical) structure was found for WWWKYE21, but substantially less so for KYE21 (Fig. 4). Due to potential contributions also from the LPS carbohydrate residues to the CD signal, detailed quantification of peptide secondary structure in the LPS complexes is precluded. Nevertheless, it is clear that the α-helix contribution dominates the CD spectra after LPS complexation, while the corresponding β-strand content was less than 1%. Such large-scale conformational changes have previously been reported for anti-inflammatory peptides^8, 9, 20^, and there correlated to peptide-induced disruption/densification of LPS aggregates, as well as to anti-inflammatory effects. In order to elucidate the peptide conformation in aqueous solution and in LPS complexes in more detail, NMR experiments were performed. Supporting the findings obtained by CD, both peptides in aqueous solution are characterized by the presence of sequential as well as intra-residue NOEs in the NOESY spectrum, indicating the peptides do not adopt any folded conformation in aqueous solution. To obtain further information on peptide conformations in LPS complexes, trNOESY experiments were performed, as almost all proton resonances of each of the peptides were found to be significantly broadened without affecting the chemical shift change in the presence of LPS (data not shown). These data clearly indicate that the peptides undergo a fast conformational exchange between free and LPS-bound form at an NMR time scale^21^.

**Figure 5:**
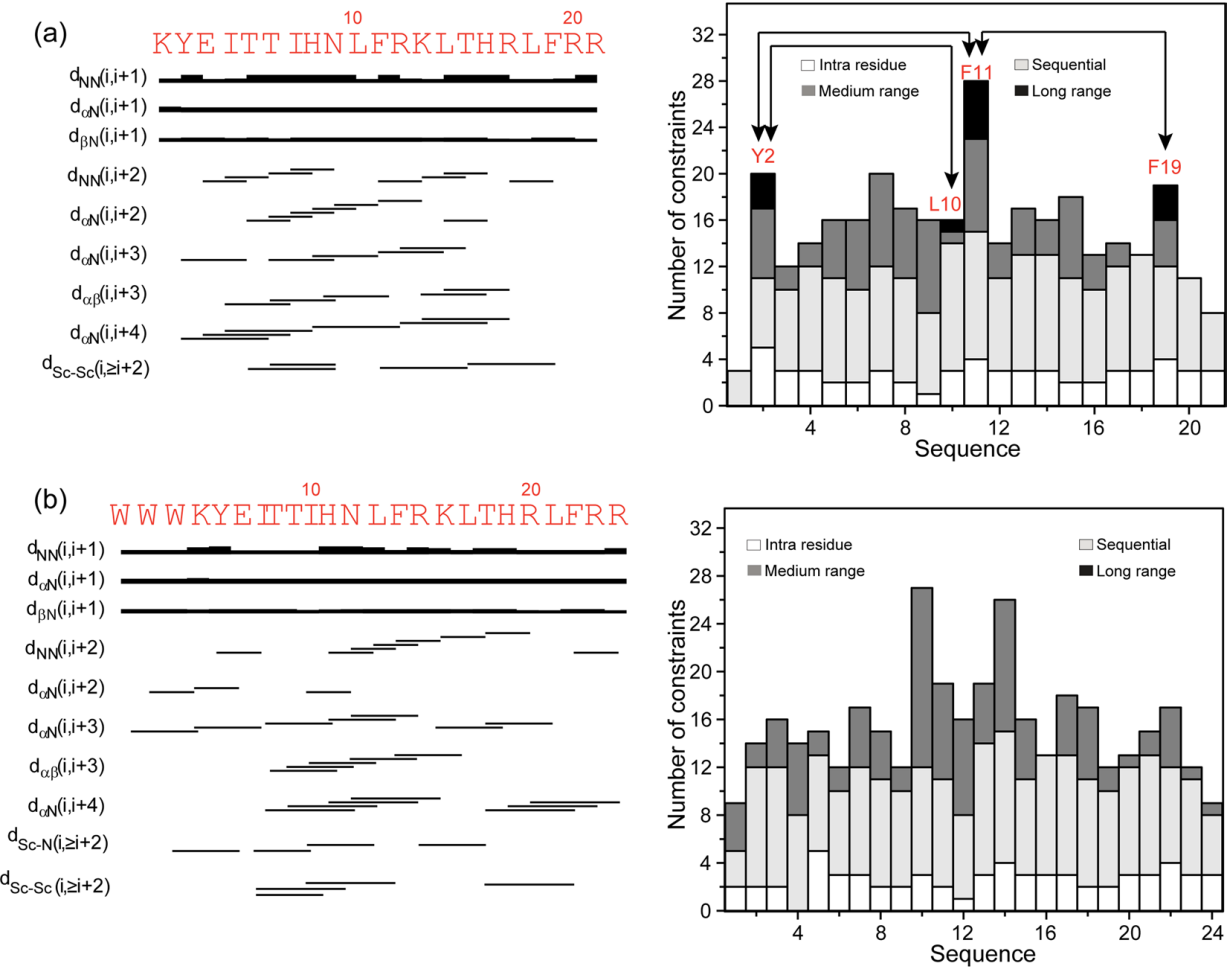
**Structural parameters of KYE21 and WWWKYE21 in LPS**. Bar diagram (left panel) and histogram (right panel) of (**a**) KYE21 and (**b**) WWWKYE21, showing important long-range and medium-range αN (i, i + 2/i + 3/i + 4), as well as long range-NOE contacts (i, ≥i + 5) among the backbone-backbone and backbone-side chain resonances found in the trNOESY spectra, used to calculate the bound conformation of the peptides. The bar thickness in the bar diagrams corresponds directly to the NOE intensities. The primary amino acid sequences are provided at the top of the bar diagrams. The histogram depicts the number of NOE constraints used to calculate the bound conformation of the peptides as a function of residue number. The arrowheads in the histogram mark the residues that share long-range contacts with each other.

The trNOESY spectra of KYE21 (Fig. 5a, left panel) and WWWKYE21 (Fig. 5b, left panel) in LPS showed several medium-range αN (i, i + 3/i + 4), NN (i, i + 2),αβ (i, i + 3), side chain-NH (i, ≥ i + 2), and side chain-side chain (i, ≥i + 2) NOE contacts, apart from the sequential NN (i, i + 1) and αN (i, i + 1) contacts, signifying the formation of a helical structure. In case of KYE21, the medium-range NOE contacts αN (i, i + 3/i + 4) were found to occur consecutively from I4 to R17, indicating the presence of a significant helical conformation in this stretch. In a similar fashion, WWWKYE21 showed NOE contacts for the residues W3 to R24 (“Supplementary Fig. S1”). It is to be noted that the chemical shifts of the aromatic ring protons and the amide protons of the aromatic residues in both peptides were well separated, thus allowing unambiguous assignments. In case of KYE21, long-range NOE contacts were observed between aromatic ring protons of Y2/F11, F11/F19, and between side chain protons and aromatic ring protons of Y2/L10 (Fig. 5a, right panel) (“Supplementary Table S1 and Fig. S1”). Strikingly, no long-range NOE contacts were observed for WWWKYE21 (Fig. 5b, right panel). However, several medium-range NOE contacts were observed, such as I10/F14 and T18/F22, as well as aromatic-aromatic NOE contacts between W1/W3, W2/W3 and W3/Y5 (“Supplementary Table S1”). Several aromatic residues W1, W2, W3, and Y5 surround K4 and hence the aliphatic side chains of K4 were shifted up-field. Notably, the chemical shifts of the two peptides were significantly different despite the sequence similarity.

**Figure 6:**
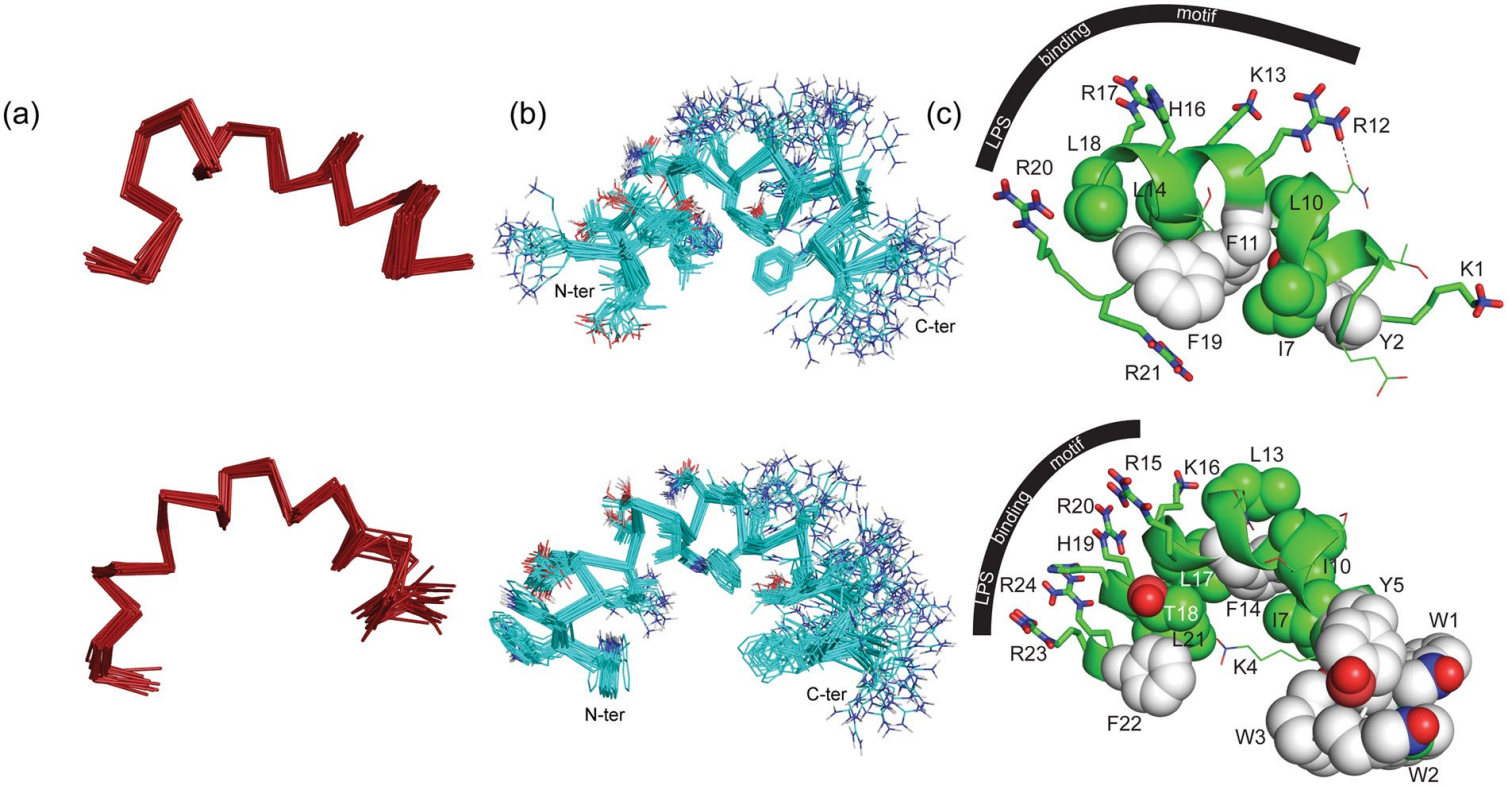
**NMR-derived three-dimensional structures of KYE21 (pdb acquisition code: 2NCU) and WWWKYE21 (PDB acquisition code: 2NCW) in LPS micelles**. (**a**) Ribbon representation of the ensemble of 20 lowest energy structures of KYE21 (upper panel) and WWWKYE21 (lower panel), showing a superimposition of the backbone (Cα, N and C′) atoms. (**b**) Ribbon and line representation of the 20 lowest energy ensemble structures of KYE21 (upper panel) and WWWKYE21 (lower panel), showing the superimposition of the backbone and the side chain atoms. (**c**) Cartoon representation of the orientations of the single structures of KYE21 (upper panel) and WWWKYE21 (lower panel) bound to LPS micelles, with aromatic (white color) and hydrophobic residues participating in the hydrophobic hub of the molecule indicated. The side chains of the arginine, lysine, and histidine residues participating in the polar face are shown in stick representation. All figures have been prepared using PyMOL.

The three-dimensional NMR structure of the peptides, derived using these NOE contacts, showed that both peptides had a well-defined structure in LPS, as evident from the close superimposition of the twenty lowest energy ensemble structures (Fig. 6a), showing a well conserved backbone and side chain, as also evident from the low RMSD values (“Supplementary Table S2”). In contrast, the flexible side chains of the positively charged arginine and lysine residues (Fig. 6b) or the fairly flexible C-terminus showed higher RMSD values (Fig. 6b). KYE21 was characterized by an extended C-terminus, followed by a helical stretch, wherein Y2/F11 and F11/F19 came close to each other driving hydrophobic stabilization (Fig. 6c, upper panel). The central region of the α-helix of KYE21 was bent due to presence of β-branched “N9-L10-F11” residues. Residues I7/L10/L14/L18 governed the i to i + 3/4 side chain-side chain hydrophobic interaction, additionally stabilizing the structure. H16 also shared the polar face probably being involved in forming initial facile contacts with the negatively charged phosphate head groups of LPS (Fig. 6c, upper panel).

WWWKYE21 was fairly dissimilar to KYE21 in being almost completely α-helical in nature, with a rigid N-terminus and a moderately flexible C-terminus (Fig. 6b, lower panel). The flexibility observed at the side chains of the C-terminus is probably due to the presence of the dynamic positively charged arginine residues responsible for initiating plausible salt-bridge or hydrogen bonding interactions with the negatively charged phosphate head groups of LPS. Lack of long-range NOE between the aromatic residues located at the two ends of the peptide, in turn, could be due to steric hindrance and may also have led to flexibility of the C-terminus. The aromatic tryptophan and tyrosine residues at the N-terminus were key in stabilizing the structure, forming an aromatic tetrad through hydrophobic and π − π stacking interactions. The other aromatic and hydrophobic residues I7/I10/L13/F14/L17/T18/F22 interacted with each other to form a hydrophobic face of the helix with the arginine, lysine, and histidine residues being oriented in the opposite direction, forming the polar face (Fig. 6c, lower panel).

### Anti-inflammatory effect and cell toxicity

**Figure 7:**
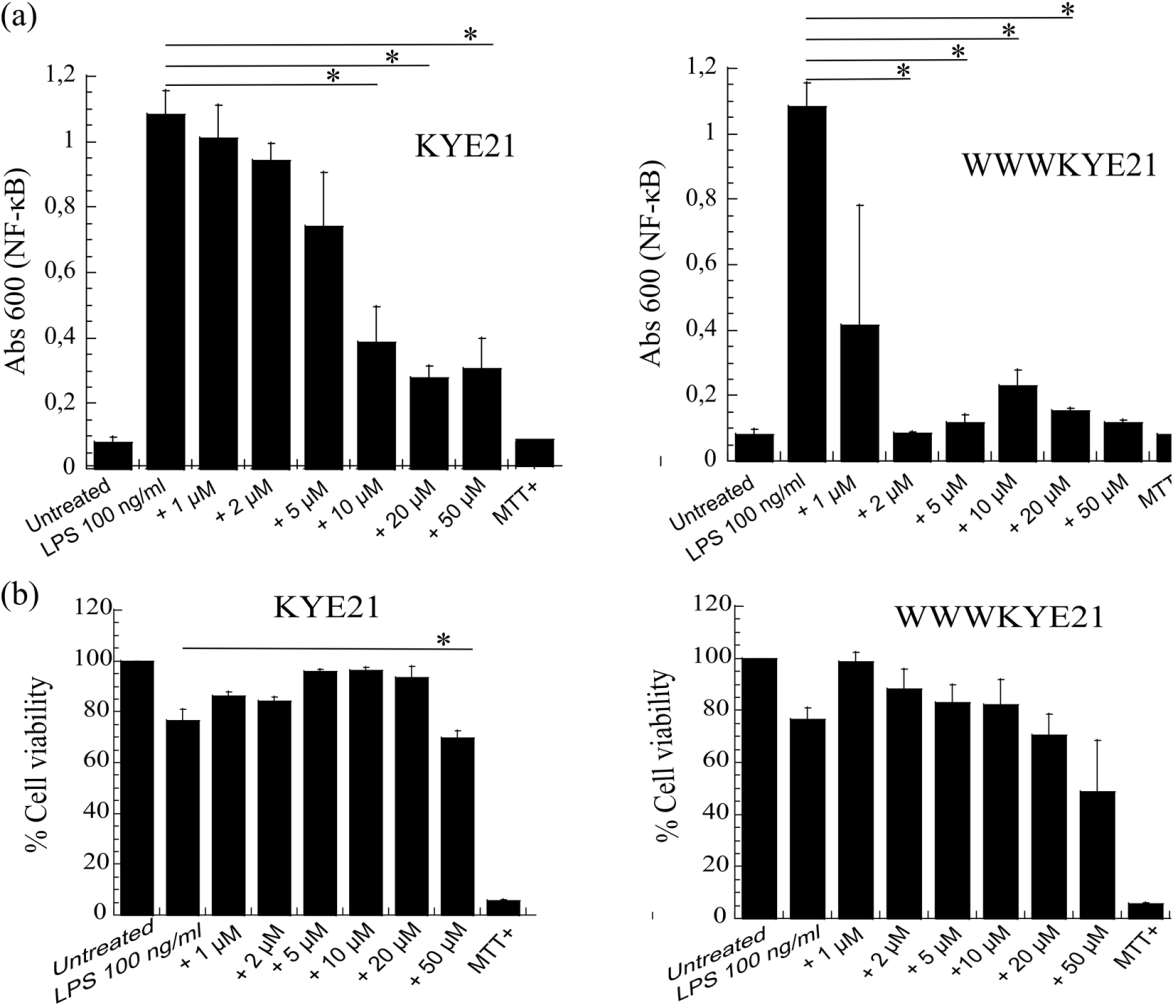
(**a**) Effect of the indicated peptides on human monocytes. THP1-XBlue-CD14 monocytes were incubated with *E. coli* LPS in presence of peptides at the indicated concentrations, followed by monitoring of NF-κΒ activation. (**b**) MTT results on peptide toxicity, demonstrating that THP1-XBlue-CD14 receptor monocytes were not damaged by the peptides up to a peptide concentration of 20–50 μM. (n = 3; mean ± SD shown) *p < 0.05 compared to absence of peptide.

**Figure 8:**
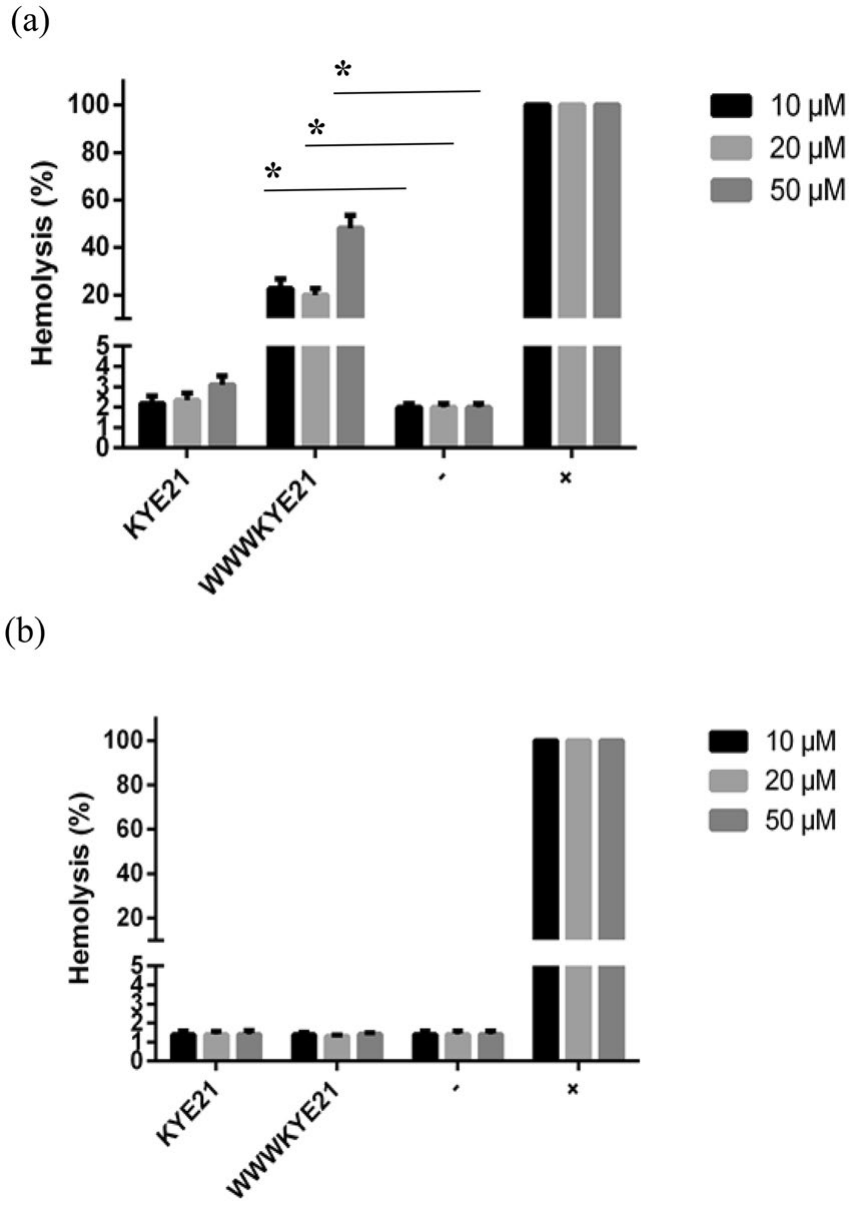
Hemolysis of erythrocytes in PBS (**a**) and 50% citrate blood (**b**) of KYE21 and WWWKYE21 at the indicated concentrations. Shown also are results from positive (+) and negative (−) controls. (n = 3; mean ± SD shown) *p < 0.05 compared to absence of peptide.

In order to evaluate to what extent the increased LPS and lipid A binding displayed by WWWKYE21 contribute to its anti-inflammatory properties, the ability of the peptides to block LPS-induced NF-κB activation reaction in human monocytes was investigated. As shown in Fig. 7a, WWWKYE21 displays potent enhancement with regards to suppression of LPS-induced NF-κB activation compared to KYE21. Quantitatively, complete suppression of NF-κB activation is observed already at a peptide concentration of 2 µM. Correspondingly, LPS induced apoptosis was inhibited by the peptide at doses of 1–2 µM ^22^. In comparison, similar suppression of NF-κB activation is not seen even at 50 µM in the case of KYE21. Strikingly, this dramatic boosting of anti-inflammatory (as well as antimicrobial) effects does not come at the prize of substantially increased peptide toxicity. As shown by MTT results for THP1-XBlue-CD14 monocytes, WWWKYE21 displays similar low toxicity as KYE21, with little or no detectable toxicity up to a peptide concentration of at least 20 µM, i.e., 10x higher than the peptide concentration needed for complete suppression of NF-αB activation for the same cells (Fig. 7b). Supporting this, hemolysis data shows that while hemolysis increases after W-tagging for erythrocytes suspended in PBS (Fig. 8a), it remains very low, at the same level as the negative control, for erythrocytes in 50% blood, up to a peptide concentration of 50 µM (Fig. 8b). In order to further demonstrate the antimicrobial selectivity of the peptides under biologically relevant conditions, we performed another set of experiments, in which *E. coli* (10^4^ cfu/ml) was added to 50% blood, simultaneously monitoring both bacterial killing and hemolysis in the same samples. As demonstrated in Supplementary Fig. S2, although both KYE21 and WWWKYE21 display some minor increase in hemolysis compared to those of the negative control and of bacteria-loaded blood in the absence of peptide, hemolysis remains very low (<3.6 ± 0.5%) also at a peptide concentration of 100 µM. At the same concentration, both peptides efficiently eradicate (>98%) blood-localized *E. coli* in the same samples. This clearly demonstrates that both peptides display a pronounced selectivity of bacteria over erythrocytes.

### Mode-of-action

**Figure 9:**
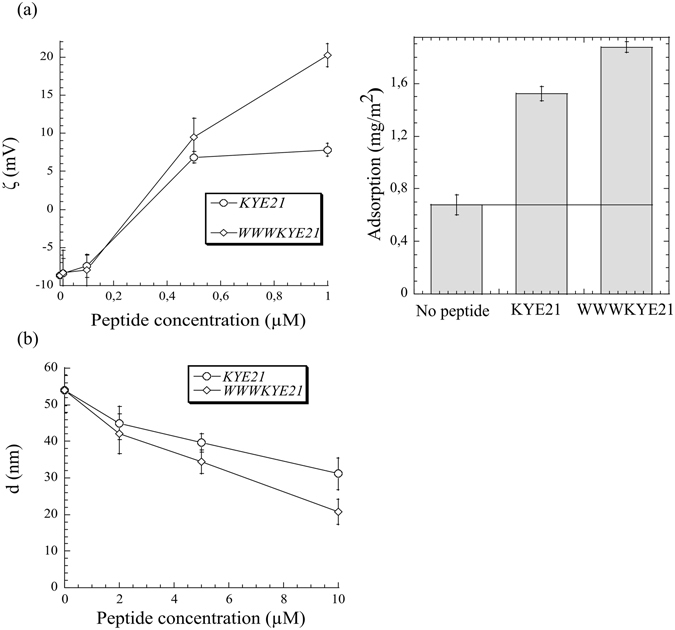
(**a**) Z-potential of DOPC/cholesterol (60/40 mol/mol) liposomes as a function of peptide concentration in 10 mM Tris, pH 7.4 (left), as well as LPS binding to DOPC/cholesterol bilayers after peptide pre-adsorption from 1 µM (right). (**b**) Mean aggregate diameter for LPS-peptide complexes for KYE21 and WWWKYE21 in 10 mM Tris, pH 7.4. Measurements were performed at a fixed LPS concentration of 0.2 mg/ml, varying the peptide concentration. (n ≥ 2).

Previously, three mechanisms of the anti-inflammatory effects of KYE28 and KYE21 were identified, i.e., (i) direct binding to LPS/lipid A and prevention of LBP binding, (ii) peptide-induced charge reversal of mammalian cell membranes for LPS scavenging, and (iii) fragmentation of LPS aggregates for improved phagocytosis, the two latter providing alternatives to the NF-κB-generating pathway^9^. Addressing the effects of W-tagging of KYE21 regarding this demonstrates that WWWKYE21 displays increased binding to both LPS and lipid A (Fig. 3), as well as enhanced peptide-induced charge reversal of mammalian-mimicking DOPC/cholesterol membranes, and enhanced LPS binding to peptide-saturated membranes resulting from this (Fig. 9a). Here, it should be noted that binding of KYE21 and WWWKYE21 to DOPC/cholesterol liposomes does not cause any significant change in liposome size, hence flocculation is not an issue in these systems. However, both KYE21 and WWWKYE21, and particularly the latter, induce leakage from these liposomes, effects previously demonstrated to be due to peptide insertion into the polar head group and resulting curvature strain^23, 24^, WWWKYE21 more so than the non-tagged KYE21 (“Supplementary Fig. S3”), the latter in agreement with the hemolysis observed in PBS (Fig. 8a). Furthermore, peptide-induced LPS micelle disintegration is more pronounced for WWWKYE21 than for KYE21, most clearly seen at a peptide concentration of 10 µM (Fig. 9b). Taken together, these results indicate that W-tagging does not change the mode-of-action qualitatively, but rather accentuates all these effects quantitatively.

## Discussion

As for hydrophobic point mutations, hydrophobic acylation promotes membrane binding and antimicrobial activity of AMPs, increasing with the length of the hydrophobic moiety^25^. Due to the long alkyl chains of commonly investigated lipopeptides, these insert into lipid membranes essentially independent of membrane composition^26^. Such lipopeptides therefore display considerable toxicity, restricting them to local use, and to severe indications for which other antibiotics are ineffective^25^. In contrast, end-tagging with aromatic W and F stretches have been found to result in high, but *selective*, antimicrobial activity^18, 19^. As with other antimicrobial peptides, the antimicrobial effect of W-tagged peptides is due to bacterial membrane lysis^19^. Through the hydrophobic interactions provided by the W residues, peptide binding to, and destabilization of, phospholipid membranes are not dramatically suppressed at high ionic strength. The origin of these effects is that bulky W and F residues require substantial area expansion for membrane insertion, which is energetically costly for membranes containing cholesterol. Together with membrane charge differences, this results in a pronounced selectivity between bacteria and human cells^19^ (Figs 2, 8, and S2). Favourable from a drug development perspective, W/F-tagging may be used to obtain selective ultra-short AMPs, displaying potency and salt resistance down to 4-7 amino acids^27^. Also difficult pathogens are counteracted by W-tagged peptides. For example, RRPRPRPRPWWWW-NH_2_ was found to be effective against a range of superbugs, including methicillin-resistant *S. aureus* (MRSA), multi-drug resistant *P. aeruginosa*, and vancomycin-resistant enterococci, but still displaying low toxicity^28^. Based on results from two different cell types (human monocytes and erythrocytes) in combination with two different toxicity assays (MTT and hemolysis), the latter selectivity seems to apply also to the presently investigated peptides, as most clearly seen for *S. aureus* (Fig. 2b). Furthermore, MTT assay on monocytes shows no signs of toxicity at a peptide concentration of at least 10 μM (Fig. 7b), which is 5 times higher than the peptide concentration required for maximal NF-κB suppression (2 μM) (Fig. 7a). Hemolysis further supports this, indicating no hemolysis above that of the negative control in 50% blood, even at 50 μM, i.e., at a 25 times higher peptide concentration that that required (2 μM) for maximal NF-κB suppression (Fig. 8). Potent bacterial killing in *E. coli*-loaded 50% citrate blood at simultaneously less than 4% hemolysis in the same samples (“Supplementary Fig. S2”) further demonstrates this selectivity. Having said that, we do note onset of some toxicity of WWWKYE21 in the artificial environment of PBS, as well as peptide-induced lysis of DOPC/cholesterol liposomes in 10 mM Tris buffer. In the presence of blood/serum, however, conditions more relevant for the situation *in vivo*, clear selectivity is observed between cell toxicity, on one hand, and antimicrobial and anti-inflammatory effects, on the other. For further development of these peptide into potential therapeutics, however, further toxicity assessments are needed, depending on the intended application.

Analogously, W-tagging may provide a tool for increasing selective anti-cancer effects, as demonstrated by pronounced peptide internalization in melanoma cells, resulting in toxicity against these, but not against the non-malignant cells^29^. From a combination of biophysical studies on membrane binding/destabilization and biological studies on cell uptake and toxicity, these effects were shown to be due to increased peptide adsorption to the outer membrane in melanoma cells, caused by the presence of anionic lipids such as phosphatidylserine and ganglioside GM1, and to peptide effects on mitochondria membranes and resulting apoptosis. In addition, W-tagging could be used for achieving targeted uptake of nanoparticles/drug carriers in melanoma, as well as for facilitating uptake of the low Mw anticancer drug doxorubicin.

W-tagging may be used to enhance peptide activities also in other context than membrane disruption. Thus, focusing on receptor interactions, Ember *et al*. found that hydrophobic tagging increased the biological potency of short C3a-derived peptides, an effect increasing with the number of terminal W residues^30^. Thus, W-tagging promoted specific peptide binding to the C3a receptor, demonstrating this approach to have potential to boost also biological effects more broadly. Considering this, we hypothesized that W-tagging could be used also for promoting anti-inflammatory effects of peptides. It has previously been demonstrated that peptide interactions with LPS depends on peptide hydrophobicity. For example, Rosenfeldt *et al*. found binding of acylated K/L peptides to LPS-containing liposomes to increase with increasing peptide hydrophobicity^31^. The importance of combined hydrophobic and electrostatic interactions has also been reported. For example, Andrä *et al*. reported on hydrophobically and electrostatically driven LPS binding of NK-2^32^, while Singh *et al*. reported analogous results for a series of S1 peptides^20^. In a subsequent study, Singh *et al*. found pronounced effects of peptide linear amphiphilicity on LPS and LTA binding of GKY25 peptide variants^33^. Thus, it is relatively well established that anti-inflammatory effects can be enhanced by increasing peptide hydrophobicity, charge, or combinations thereof. However, as discussed above, hydrophobic modifications almost invariably comes at the prize of pronounced toxicity, while electrostatic effects are strongly attenuated at high ionic strength^10, 26^. In contrast, W-tagging allows both anti-inflammatory and antimicrobial effects to be potently retained at physiological ionic strength, yet keeping cell toxicity low, here demonstrated for both monocytes and erythrocytes.

Although a broader comparison of the biological effects of WWWKE21 and KYE21 has not been performed, e.g., relating to cell internalization, phagocytosis-induction of LPS, and other cellular processes, biophysical model experiments, in combination with data on anti-inflammatory effect, indicate that W-tagging does not dramatically change the mechanisms behind the anti-inflammatory effects. Instead, W-tagging accentuate mechanisms present also for KYE21, notably direct binding to lipid A (with implications from LBP binding), but also peptide-induced charge reversal of mammalian cell membranes for LPS scavenging, and fragmentation of LPS aggregates for improved phagocytosis. In relation to the latter mechanism, it has been previously demonstrated by cryoTEM that LPS aggregates are poorly defined, displaying thread-like structures, branching, and polydispersity/heterogeneity. In the presence of KYE21, however, LPS is disintegrated to form smaller, more spherical, and more uniform particles^9^. Such peptide-induced LPS aggregate disintegration (Fig. 9b and “Supplementary Fig. S4”), as well as direct lipid A binding (Fig. 3b), and peptide-induced membrane-localized membrane scavenging of LPS (Fig. 9a, “Supplementary Fig. S5”), seems to be promoted by W-tagging. Although the relative importance of these different modes-of-action need to be substantiated through further studies, notably on peptide-induced charge reversal of macrophages (in the absence of potential colloidal scale structural effects present for liposomes) and peptide-induced LPS endocytosis, these findings suggests that W-tagging does not qualitatively change the mode-of-action of KYE21, in turn opening up for generalization of the approach to other peptides.

Having said that, there is also limits for the beneficial effects of W-tagging. For example, Singh *et al*. investigated antimicrobial and anti-inflammatory effects of the thrombin-derived peptide GKY25 (GKYGFYTHVFRLKKWIQKVIDQFGE) and its variant WFF25 (WFFFYYLIIGGGVVTHQQRKKKKDE), of identical composition but with the amino acids sorted according to hydrophobicity. Through its pronounced linear amphiphilicity, the latter peptide forms aggregates in solution, which suppresses anti-inflammatory effects^33^. Hence, W-tagging must be balanced in relation to the individual peptide (length, hydrophobicity, charge) in order to avoid such self-assembly.

## Methods

### Chemicals

Peptides (Table 1) were synthesized by Biopeptide Co., San Diego, USA, and were of >95% purity, as evidenced by mass spectral analysis (MALDI-TOF Voyager). DOPC (1,2-dioleoyl-*sn*-Glycero-3-phosphocholine), DOPG (1,2-dioleoyl-*sn*-Glycero-3-phosphoglycerol, monosodium salt), and DOPE (1,2-dioleoyl-*sn*-Glycero-3-phosphoethanolamine) were from Avanti Polar Lipids (Alabaster, USA) and of >99% purity, while cholesterol (>99%) was from Sigma-Aldrich (St. Luis, USA). LPS from *E. coli* (0111:B4) and lipid A from *E. coli* F583 (Rd mutant) were both from Sigma (St. Louis, USA).

**Table 1:** Primary structure and key properties of the peptides investigated.

*Peptide variants*	*Sequence*	*IP* ^1^	*Z*_*net*_^2^ (*pH* 7.4)	*μH*_*rel*_^3^
*KYE21*	KYEITTIHNLFRKLTHRLFRR	11.97	+5	−0.79
*WWWKYE21*	WWWKYEITTIHNLFRKLTHRLFRR	11.73	+5	−0.804

^1^IP: isoelectric point; ^2^Z_net_: net charge; ^3^ *μ*H_rel_: relative hydrophobic moment on the Kyte-Doolittle scale^45^.

### Microorganisms

*Escherichia coli* (*E. coli*) ATCC 25922 and *Staphylococcus aureus* (*S. aureus*) ATCC 29213 were obtained from the Department of Clinical Bacteriology at Lund University Hospital, Sweden.

### Antibacterial assays

Minimum inhibitory concentration (MIC) determinations reflect bacterial inhibition, and antimicrobial peptides may also be affected by media components^34^. Therefore, in order to evaluate direct bacterial killing in defined environments, viable count analysis (VCA) was chosen for monitoring antimicrobial effects, combined with radial diffusion assay (RDA) measurements as a second assay for redundancy.

#### Radial diffusion assay

Bacteria (*E. coli* and *S. aureus*) were grown to mid-logarithmic phase in 10 mL of 3% w/v trypticase soy broth (TSB) (Becton-Dickinson, Cockeysville, MD). The microorganisms were then washed with 10 mM Tris, pH 7.4, 0.15 M NaCl. After this, 6 × 10^6^ bacterial colony forming units were added to 15 mL of the underlay agarose gel, consisting of 1% (w/v) low electro-endosmosis type (low-EEO) agarose, 0.02% (v/v) Tween 20 (both Sigma- Aldrich) and 0.03% (w/v) TSB. The underlay formed by the latter was poured into Ø 150-mm petri dishes. After agarose solidification, 4 mm diameter wells were punched in the underlay and 6 µL of test sample was added to each well. Plates were then incubated at 37 °C for 3 hrs to allow peptide diffusion. After this incubation time, the underlay gel was covered with 15 mL of molten overlay (6% TSB and 1% low-EEO agarose in dH_2_O), and the peptide antimicrobial activity was visualized as clear zones around each well after 18–24 hrs of incubation at 37 °C. Data presented from triplicate experiments in the peptide concentration range 10–100 μM are expressed in term of mean diameter of the clear zones formed for the different peptides.

#### Viable-count analysis

Bacteria (*E. coli* and *S. aureus*) were grown to mid-logarithmic phase in Todd-Hewitt (TH) medium. Bacteria were washed and diluted in 10 mM Tris, pH 7.4, 5 mM glucose, 0.15 M NaCl. Bacteria (50 µL; 2 × 10^6^ bacteria/ml) were then incubated at 37 °C for 2 h in the presence of peptide at the indicated concentrations. Serial dilutions of the incubation mixture were plated on TH agar, followed by incubation at 37 °C overnight, after which the number of colony-forming units (cfu) was determined. Data shown, obtained at peptide concentrations of 10, 25, and 50 μM, are mean values obtained from triplicate measurements.

### LPS effects on human monocytes *in vitro*

THP1-XBlue-CD14 reporter cells (1 × 10^6^ cells/mL) (InvivoGen, San Diego, USA) were seeded in phenol red RPMI (180000 cells/well), supplemented with 10% (v/v) heat-inactivated FBS and 1% (v/v) AAS, and allowed to adhere before they were stimulated with 100 ng/mL *E. coli* (0111:B4) LPS and with peptides at the indicated concentrations. NF-κB activation was determined after 20 h of incubation according to manufacturer’s instructions (InvivoGen, San Diego, USA). Briefly, activation of NF-κB leads to the secretion of embryonic alkaline phosphatase (SEAP) into the cell supernatant, were it was measured by mixing supernatant with a SEAP detection reagent (Quanti-Blue^TM^, InvivoGen), followed by absorbance measurement at 600 nm. Data shown in the peptide concentration range 0–50 μM are mean values obtained from triplicate measurements.

### MTT assay

Sterile filtered MTT (3-(4,5-dimethylthiazolyl)-2,5-diphenyl-tetrazolium bromide; Sigma-Aldrich) solution (5 mg/mL in PBS) was stored protected from light at −20 °C until usage. THP1-XBlue-CD14 cells, 180000 cells/well, were seeded in 96 well plates and grown in phenol red RPMI, supplemented with 10% (v/v) heat-inactivated FBS and 1% (v/v) AAS to confluency. Peptides investigated were then added at 1–50 µM. After incubation over night, 20 µL of the MTT solution was added to each well and the plates incubated for 1.5 h in CO_2_ at 37 °C. The MTT-containing medium was then removed by aspiration. The blue formazan product generated was dissolved by addition of 100 µL of 100% DMSO per well, and the plates gently swirled for 30 min at room temperature to dissolve the precipitate. The absorbance was monitored at 550 nm, and results given in the peptide concentration range 0–50 μM represent mean values from triplicate measurements.

### Hemolysis assay

EDTA-blood was centrifuged at 800 g for 10 min, whereafter plasma and buffy coat were removed. The erythrocytes were washed three times and re-suspended in PBS, pH 7.4, to a 5% suspension. The cells were then incubated with end-over-end rotation for 1 h at 37 °C in the presence of peptides (0–50 μM). 2% Triton X-100 (Sigma-Aldrich) served as positive control. In another experiment, whole citrated blood was diluted 1:1 in PBS and incubated with the peptides at indicated concentrations for 1 h 37 °C. In all cases, the absorbance of hemoglobin release was measured at λ 540 nm and is expressed as % of Triton X-100-induced hemolysis in the peptide concentration range 0–50 μM. In the experiments with blood infected by bacteria, citrate-blood was diluted (1:1) with PBS. The cells were then incubated with end-over-end rotation for 1 h at 37 °C in the presence of peptides (100 µM) and *E. coli* (10^4^ cfu/ml) bacteria. For evaluation of hemolysis, samples were then processed as above, while bacterial killing was monitored as in the viable count assay, also described above. All methods were carried out in accordance with relevant guidelines and regulations. The use of human blood was approved by the Ethics Committee at Lund University, Lund, Sweden (Permit Number: 657-2008), and informed consent was obtained from all subjects.

### Liposome preparation and leakage assay

The lipid mixture (DOPE/DOPG, 75/25 mol/mol) was dissolved in chloroform, after which solvent was removed by evaporation, first under vacuum at 60 °C for 45 minutes, and then in a vacuum oven (Lab-line, Melrose Park, NJ, USA) under vacuum at room temperature overnight. Subsequently, 10 mM Tris buffer, pH 7.4, was added together with 0.1 M carboxyfluorescein (CF) (Sigma, St. Louis, USA). After hydration, the lipid mixture was subjected to eight freeze-thaw cycles, consisting of freezing in liquid nitrogen followed by heating to 60 °C and vortexing. Unilamellar liposomes of about ϕ140 nm were generated by multiple extrusions (30 passages) through polycarbonate filters (pore size 100 nm) mounted in a LipoFast miniextruder (Avestin, Ottawa, Canada) at 22 °C. Untrapped CF was removed by two subsequent gel filtrations (Sephadex G-50, GE Healthcare, Uppsala, Sweden) at 22 °C, with Tris buffer as eluent. Leakage from liposomes was studied by monitoring reduction of CF self-quenching upon release from the liposomes interior by using Spex fluorolog 1680 0.22 m double spectrometer (Instruments S. A. Group, Edison, NJ, USA). The emitted fluorescence from the liposome dispersion (10 µM lipid in 10 mM Tris, pH 7.4) was followed at 520 nm. For leakage experiment, peptide was added to the liposomes and leakage monitored as a function of time. An absolute leakage scale was obtained by disrupting the liposomes at the end of each experiment through addition of 0.8 mM Triton X-100 (Sigma-Aldrich, St. Louis, USA). As generally found for potent antimicrobial peptides, liposome leakage induced by KYE21 and WWWKYE21 is fast, being essentially completed in less than 5 minutes (“Supplementary Fig. S6”). Measurements in the peptide concentration range 0–1 μM were performed in at least duplicate at 37 °C.

### Ellipsometry

Peptide adsorption was studied *in situ* by null ellipsometry^35, 36^, using an Optrel Multiskop (Optrel, Kleinmachnow, Germany) equipped with a 100 mW Nd:YAG laser (JDS Uniphase, Milpitas, USA). All measurements were carried out at 532 nm and an angle of incidence of 67.66° in a 5 ml cuvette under stirring (300 rpm). In brief, by monitoring the change in the state of polarization of light reflected at a surface in the absence and presence of an adsorbed layer, the mean refractive index (n) and layer thickness (d) of the adsorbed layer can be obtained. From the thickness and refractive index, the adsorbed amount (Γ) was calculated according to:

1 $${\rm{\Gamma }}=\frac{(n-{n}_{0})}{dn/dc}d$$

where dn/dc (0.154 cm^3^/g) is the refractive index increment and n_0_ is the refractive index of the bulk solution. Corrections were routinely done for changes in bulk refractive index caused by changes in temperature and excess electrolyte concentration.

LPS-coated surfaces were obtained by adsorbing *E. coli* LPS to methylated silica surfaces (surface potential −40 mV, and contact angle 90°)^37^ from 5 mg/mL LPS stock solution in water at a concentration of 0.4 mg/mL in 10 mM Tris, pH 7.4. This LPS concentration corresponds to saturation in the LPS adsorption isotherm under these conditions. Non-adsorbed LPS was removed by rinsing with Tris buffer at 5 ml/min for a period of 30 minutes, allowing the buffer stabilization for 20 minutes. Peptide addition was subsequently performed to 0.01, 0.1, 0.5, and 1 µM, and adsorption monitored for at least one hour after each addition. All measurements were performed in at least duplicate at 25 °C.

For lipid A deposition, this was solubilized with 0.25 wt% triethyl amine (TEA) under vigorous vortexing and heating to 60 °C for 10 minutes^38^. Lipid A was adsorbed at methylated silica surfaces for 2 hours from 5 mg/ml lipid A stock solution in 0.25% TEA at a concentration of 0.4 mg/mL in 10 mM Tris, pH 7.4. Non-adsorbed lipid A was subsequently removed by rinsing with same buffer at 5 mL/min for 15 minutes, further followed by buffer stabilization for 20 minutes. This results in a lipid A adsorption of 0.8 ± 0.2 mg/m^2^. Peptide addition was subsequently performed to 0.01, 0.1, 0.5, and 1 µM, and adsorption monitored for at least one hour after each addition. All measurements were performed in at least duplicate at 25 °C.

Supported lipid bilayers were generated from liposome adsorption. DOPE/DOPG (75/25 mol/mol) were prepared as described above, but the dried lipid films re-suspended in Tris buffer only with no CF present. In order to avoid adsorption of peptide directly at the silica substrate through any defects of the supported lipid layer, poly-L-lysine (M_w_ = 170 kDa, Sigma-Aldrich, St. Louis, USA) was pre-adsorbed from water prior to lipid addition to an amount of 0.045 ± 0.01 mg/m^2^, followed by removal of non-adsorbed poly-L-lysine by rinsing with water at 5 mL/min for 20 minutes^39^. Water in the cuvette was then replaced by 10 mM Tris buffer, pH 7.4, 150 mM NaCl, which was followed by addition of liposomes in buffer at a lipid concentration of 20 µM and subsequently by rinsing with buffer (5 mL/min for 15 minutes) when the liposome adsorption had stabilized in order to remove non-adsorbed and weakly adsorbed liposomes. The final layer formed had structural characteristics (thickness 4 ± 1 nm, mean refractive index 1.47 ± 0.03) suggesting that a layer fairly close to a complete bilayer is formed.

For the experiment on peptide-induced LPS adsorption at mammalian membrane mimic, zwitterionic bilayers were deposited by co-adsorption from a mixed micellar solution containing 60/40 mol/mol DOPC/cholesterol and n-dodecyl-β-D-maltoside (DDM; ≥98% purity, Sigma-Aldrich, St. Louis, USA), as described in detail previously^39^. In brief, the mixed micellar solution was formed by addition of 19 mM DDM in water to DOPC/cholesterol dry lipid films, followed by stirring overnight, yielding a solution containing 97.3 mol% DDM, 1.6 mol% DOPC and 1.1 mol% cholesterol. This micellar solution was added to the cuvette at 25 °C, and the following adsorption monitored as a function of time. When adsorption had stabilised, rinsing with Milli-Q water at 5 mL/min was initiated to remove mixed micelles from solution and surfactant from the substrate. By repeating this procedure and subsequently lowering the concentration of the micellar solution, stable and densely packed bilayers are formed, with structural characteristics similar to those of bulk lamellar structures of the lipids^39^. Once formed, the bilayers obtained where equilibrated with 1 μM KYE21 or WWWKYE21 in 10 mM Tris, pH 7.4. After equilibration for 2 hours, peptides in solution were removed through rinsing with 10 mM Tris, pH 7.4 for 30 minutes, followed by adsorption of LPS at 0.4 mg/ml from 10 mM Tris, pH 7.4.

### NMR spectroscopy

All NMR experiments (one-dimensional (1D) ^1^H proton NMR titrations, two-dimensional (2D) total correlation spectroscopy (TOCSY), and transferred nuclear Overhauser effect spectroscopy (trNOESY)) were performed at 25 °C using Bruker Avance III 700 MHz spectrophotometer equipped with a 5 mm cryoprobe. Peptide and LPS samples were prepared in 10 mM phosphate buffer, pH 4.5, containing 10% D_2_O. 1D proton spectra of the peptides were recorded in aqueous solution (1 mM), followed by titration with increasing concentrations of *E. coli* O111:B4 LPS until significant line broadening effect was noted. DSS (4,4-dimethyl-4-silapentane-1-sulfonic acid) was used as an internal standard in the experiment. 2D TOCSY with a mixing time of 80 ms and trNOESY having three different mixing times (100, 150, 200 ms) were then performed on the same samples. A spectral width of 12 ppm was kept constant in both dimensions for both experiments. Sixteen dummy scans were performed while number of scans for TOCSY and trNOESY per t1 increment were set at 16 and 24, respectively. Excitation sculpting^40^ and States TPPI^41^ were used for water suppression and quadrature detection in t1 dimension, respectively. TOCSY and trNOESY spectra were zero filled and processed using 4 K (t2) × 1 K (t1) data matrices in Bruker TOPSPIN software suite. The spectra were analyzed using Sparky^42^.

Structure calculations were carried out using CYANA 2.1 software^43^ on the basis of the intensities of the cross peaks obtained from the trNOESY spectra, which were categorized into strong, medium, and weak in a qualitative manner, and converted to upper bound distances of 2.5, 3.5, and 5 Å, respectively. The bound conformations of the peptide were calculated based on trNOEs observed in their respective spectra alone. The dihedral angles ϕ and ψ were set at −30° to −120° and −120° to +120° for all residues to restrict conformational search. Hydrogen bond constraints were not used in structure calculations. Structure refinement was carried out by calculating the structure several times adjusting the NOE violations. The 20 lowest energy structures, from the 100 structures calculated, were used to represent the ensemble structure in each case. Procheck^44^ was used for the quality check of the pdb structures, while PyMOL was used for visualization.

### CD spectroscopy

Peptide secondary structure was monitored in the range 200–260 nm by circular dichroism spectroscopy (CD) using a Jasco J-810 Spectropolarimeter (Jasco, Easton, USA). The measurements were performed in a 10 mm quartz cuvette under stirring with a peptide concentration of 10 µM in 10 mM Tris, pH 7.4, in presence (0.2 mg/mL) and absence of *E. coli* LPS. To account for instrumental differences between measurements, background subtraction was performed routinely. Signals from the bulk solution were also corrected for. Measurements were performed in duplicate at 37 °C. Secondary structure quantification was performed both using the intensity at 225 nm^39^ and by applying K2D3 reference set^33^.

### Size and zeta potential measurements

Volume-averaged mean diameter of *E. coli* LPS and peptide-LPS aggregates were determined by dynamic light scattering at a scattering angle of 173°, using a Zetasizer Nano ZS (Malvern Instruments, Malvern, UK). Measurements were performed at a fixed LPS concentration at 0.2 mg/mL with varying peptide concentration in 10 mM Tris, pH 7.4. Peptide/LPS mixtures were incubated for two hours before measurements were initiated, in each case monitoring size over time to ensure absence of time-dependent effects. Using the same experimental setup, z-potential of DOPC/cholesterol (60/40 mol/mol) liposomes was measured as a function of peptide concentration (0–1 μM), and z-potential calculated using the Smulochowski approximation. In both cases, measurements were performed in duplicate at 25 °C (the latter temperature used to avoid microbubble formation in tubings and cuvette).

### Statistics

Values are reported as means ± standard deviation of the means. To determine significance, analysis of variance with ANOVA was used as indicated in the figure legends, where “n” denotes number of independent experiments. Significance was accepted at p < 0.05.

## Conclusion

Through W-tagging, WWWKYE21 displays higher membrane binding/destabilization and bacterial killing than unmodified KYE21. Analogously, WWWKYE21 binds more extensively to LPS and its lipid A moiety. In parallel, it causes more pronounced helix formation in peptide/LPS complexes than KYE21, while NMR-derived structures show formation of hydrophobically driven, more stable, peptide-LPS complexes in case of WWWKYE21. Mirroring its increased affinity for LPS and lipid A, WWWKYE21 displays strongly increased anti-inflammatory effects, while cell toxicity remained low, demonstrated both for monocytes and erythrocytes. Mode-of-action studies showed W-tagging not to change the mechanisms underlying the anti-inflammatory effect, but rather to accentuate mechanisms present also for KYE21, i.e., (i) direct binding to lipid A, (ii) peptide-induced charge reversal of mammalian cell membranes for LPS scavenging, and (iii) fragmentation of LPS aggregates for improved phagocytosis. Taken together, W-tagging thus seems to provide an interesting approach for boosting anti-inflammatory peptides, yet displaying low toxicity towards human cells.

## Electronic supplementary material

Supplementry information
