# Supplementary material for: Tryptophan end-tagging for promoted lipopolysaccharide interactions and anti-inflammatory effects
Source: Sci Rep. 2017 Mar 16;7:212. doi: 10.1038/s41598-017-00188-7 (PMC5427892; doi:10.1038/s41598-017-00188-7)
Supplement: Supplementary file 1 — Supplementry information [file 41598_2017_188_MOESM1_ESM.pdf]

# Tryptophan end-tagging for promoted lipopolysaccharide interactions and anti-inflammatory effects

Shalini Singh[1, ¶,*], Aritreyee Datta[2, ¶], Artur Schmidtchen[3,4], Anirban Bhunia[2] and Martin Malmsten[1]

[1]Department of Pharmacy, Uppsala University, SE-75232 Uppsala, Sweden

[2]Department of Biophysics, Bose Institute, P-1/12 CIT Scheme VII (M), Kolkata 700054, India

[3]Division of Dermatology and Venereology, Department of Clinical Sciences, Lund University, SE-221 84 Lund, Sweden

[4]Lee Kong Chian School of Medicine, Nanyang Technological University, 11 Mandalay Road, Singapore 308232

[¶]Contributed equally to this work

[*]Corresponding author. Tel: +46184714368; Fax: +46184717725; E-mail: shalini.singh@farmaci.uu.se

## Supporting Material

**Table S1.** A list of the important long-range and medium-range contacts in KYE21 and WWWKYE21 crucial for structural stabilization in LPS micelles.

| KYE21 | WWWKYE21 |
|---|---|
| Tyr2H3-Phe11H2 | Trp3C$^{\beta}$H-Trp1H7 |
| Leu10C$^{\beta}$H-Tyr2H2 | Trp3C$^{\beta}$H-Trp1H2 |
| Ile7C$^{\delta}$H-Tyr2H2 | Trp3C$^{\alpha}$H-Trp1H7 |
| Ile7C$^{\delta}$H-Tyr2H3 | Trp2C$^{\alpha}$H-Lys4HN |
| Ile7C$^{\gamma}$H-Tyr2H3 | Trp3H2-Tyr5H3 |
| Ile7C$^{\gamma}$H-Tyr2H2 | Trp3C$^{\alpha}$H-Trp2H2 |
| Phe11C$^{\beta}$H-Tyr2H3 | Trp2H2-Trp3H2 |
| Phe19H2-Phe11H3 | Lys4C$^{\beta}$H-Tyr5H2 |
| Phe19H3-Phe11H2 | Lys4C$^{\beta}$H-Tyr5H3 |
| Phe11C$^{\beta}$H-Phe19H3 | Lys4C$^{\beta}$H-Trp1H2 |

**Table S2.** Summary of structural statistics for the 20 lowest energy ensemble structures of KYE21 and WWWKYE21 in LPS micelles.

| **Distance restrains** | **KYE21** | **WWWKYE21** |
|---|---|---|
| Intra-residue (i–j = 0) | 56 | 65 |
| Sequential (\|i–j\| = 1) | 87 | 96 |
| Medium-range (2≤\|i–j\|≤4) | 40 | 59 |
| Long-range (\|i–j\| ≥5) | 6 | 0 |
| **Total** | 189 | 220 |
| **Angular restraints** | 40 | 46 |
| Φ | 20 | 23 |
| Ψ | 20 | 23 |
| **Distance restraints from violations (≥ 0.4Å)** | 0 | 0 |
| **Deviation from mean structure (Å)**<br>Average back bone to mean structure<br><br>Average heavy atom to mean structure | <br>0.53± 0.20<br><br>1.50± 0.35 | <br>0.57±0.17<br><br>1.39±0.23 |
| **Ramachandran plot for mean structure**[a] | | |
| % Residues in the most favourable and additionally allowed regions<br><br>% Residues in the generously allowed Region<br><br>% Residues in the disallowed region | 100<br><br>0<br><br>0 | 100<br><br>0<br><br>0 |

[a] Based on Procheck NMR

**Figure S1.** Selected region of transferred NOESY (trNOESY) spectra (mixing time=150ms) of (A) KYE21 and (B) WWWKYE21 in LPS micelle. The experiment was performed with LPS:peptide molar ratio of 1:20 and at 298K using Bruker Avance III 700 MHz spectrometer.

(a)

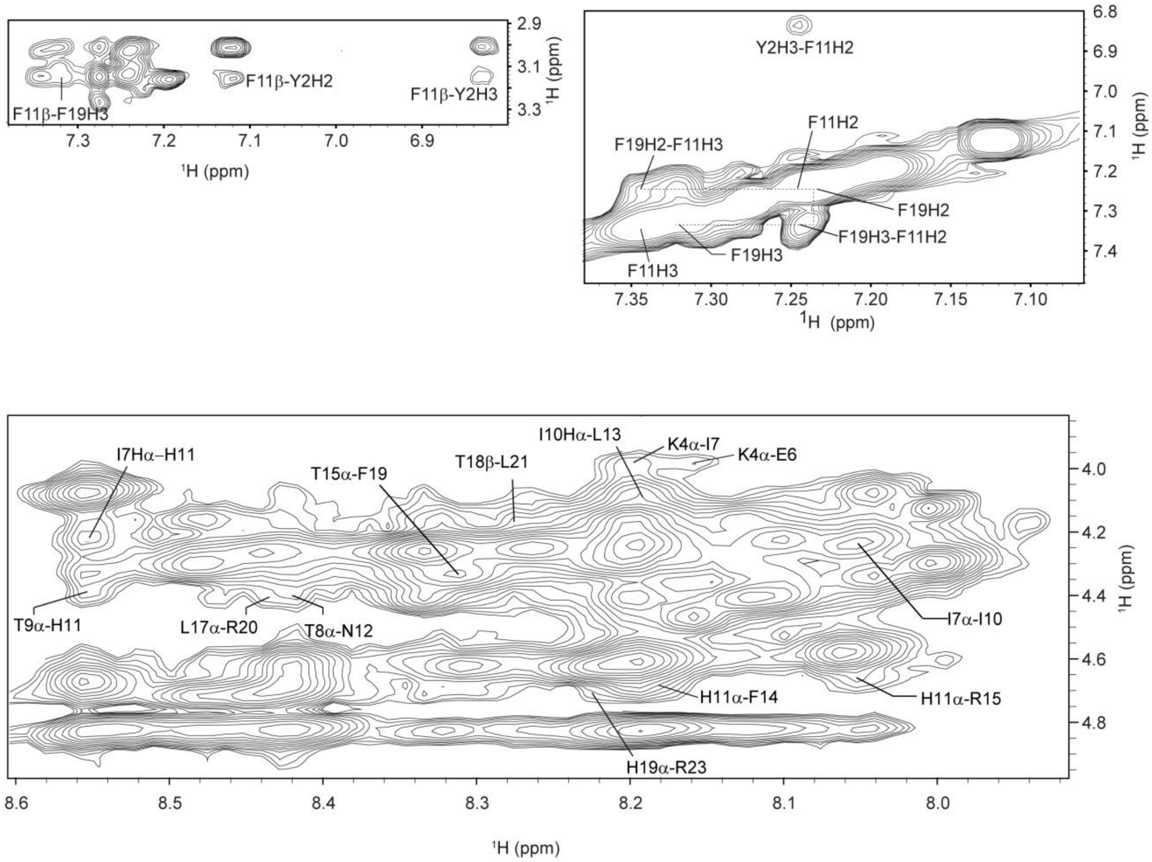

**Figure S2.** Combined hemolysis (a) and VCA assay (b) for *E. coli* ($10^4$ cfu/ml) added to 50% citrate blood, followed by exposure to 100 μM of KYE21 and WWWKYE21. As seen, although both KYE21 and WWWKYE21 display some minor increase in hemolysis compared to those of the negative control and of bacteria-loaded blood in the absence of peptide, hemolysis remains very low (<3.6±0.5%), also at a peptide concentration of 100 μM. At the same concentration, both peptides efficiently eradicate (>98%) blood-localized *E. coli* in the same samples. This clearly demonstrates that both peptides display a pronounced selectivity of bacteria over erythrocytes. (n=3; mean±SD shown) *p < 0.05 compared to absence of peptide.

(a)

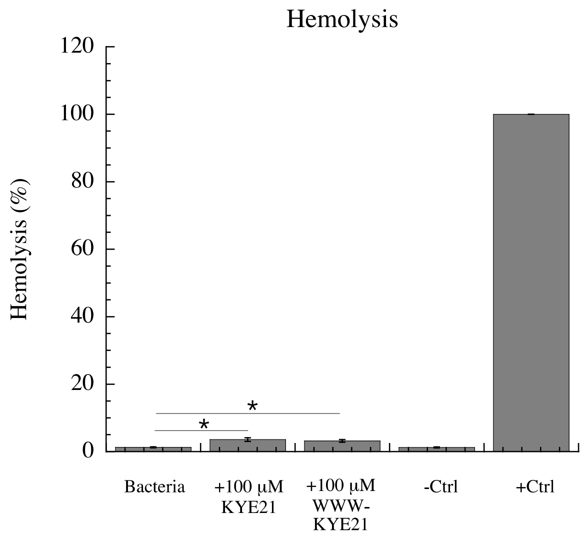


(b)

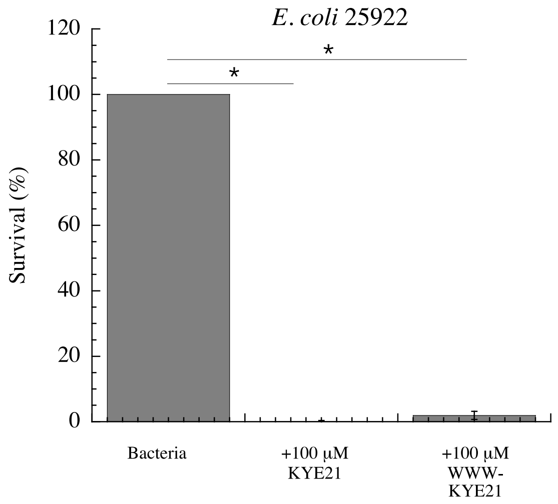

**Figure S3.** Peptide-induced liposome leakage for DOPC/cholesterol (60/40 mol/mol) in 10 mM Tris, pH 7.4.

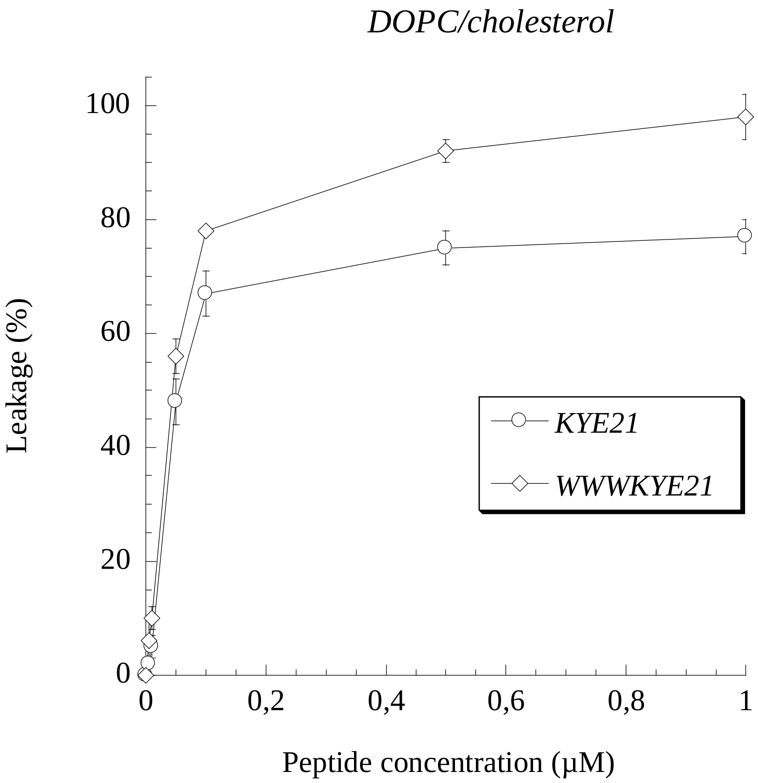

**Figure S4.** Size distributions for the LPS (0.2 mg/ml) in the absence or presence of KYE21 or WWWKYE21 at a peptide concentration of 10 µM in 10 mM Tris, pH 7.4. Insert: Expanded linear scale.

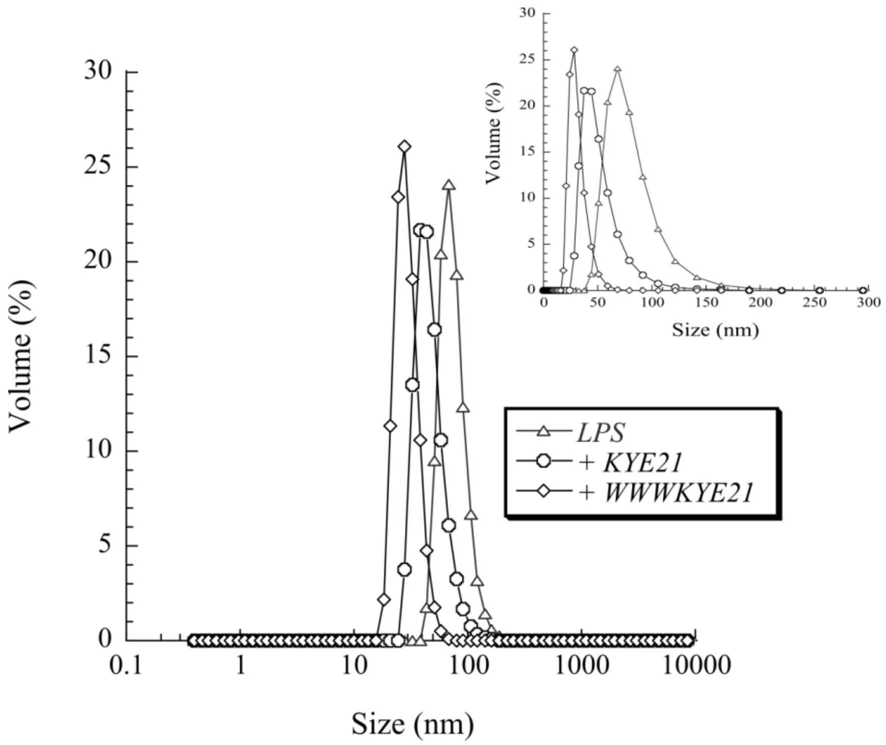

**Figure S5.** Size distribution of DOPC/cholesterol liposomes in 10 mM Tris, pH 7.4, as well as size distributions after addition of 1 μM KYE21 or WWWKYE21, respectively.

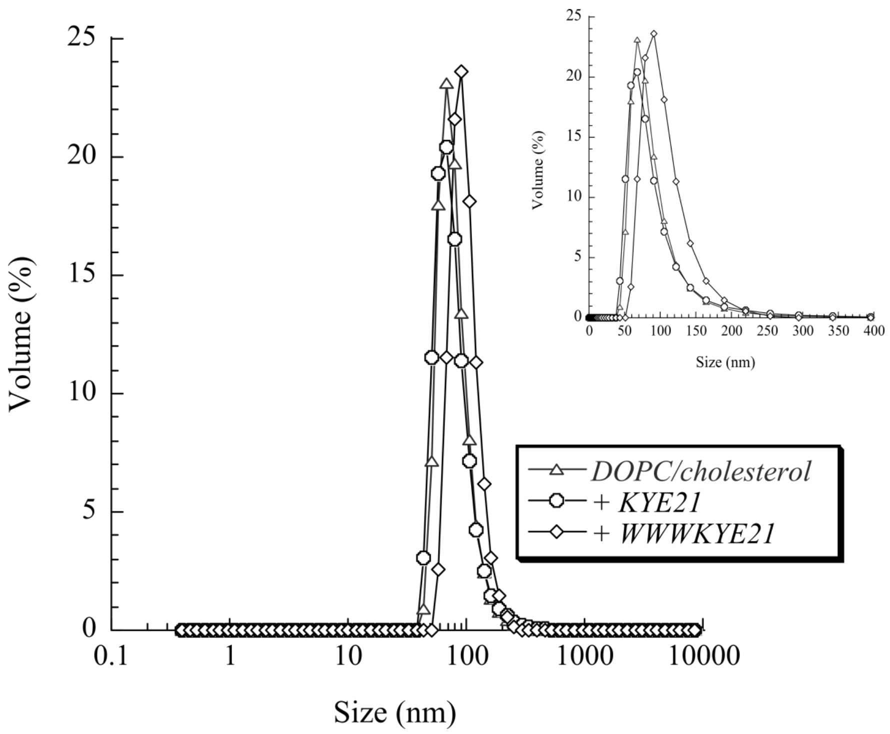

**Figure S6.** Representative peptide-induced DOPE/DOPG liposome leakage kinetics for 1 µM of peptides in 10 mM Tris, pH 7.4.

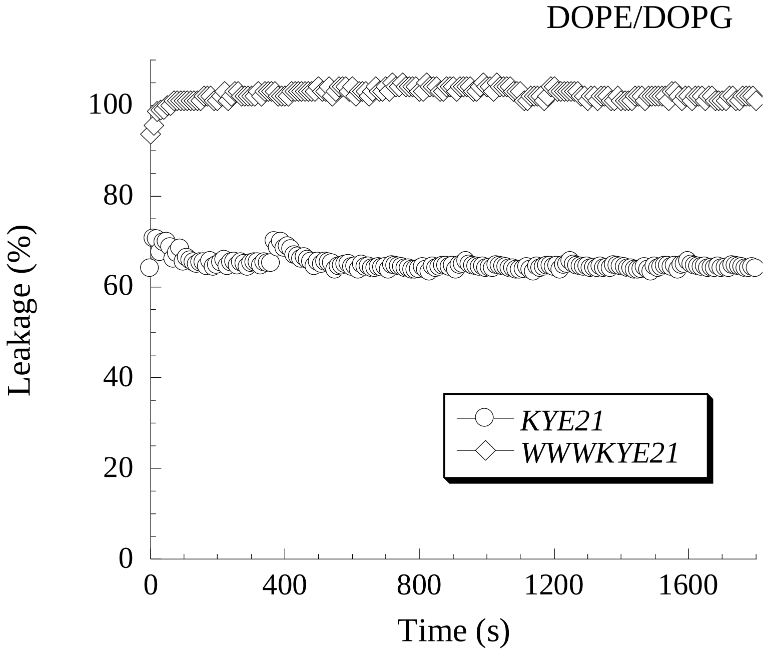

## Supplementary information

Tables containing a list of the important long- and medium-range contacts, as well as structural statistics for the 20 lowest energy ensemble structures of KYE21 and WWWKYE21 in LPS micelles. NOESY data and chemical shifts for KYE21 in LPS micelles are also included. The structures of KYE21 and WWWKYE21 bound to LPS have been deposited to protein data bank (PDB) with accession numbers 2NCU and 2NCW, respectively. Figures showing concentration-dependent leakage of DOPC/cholesterol liposomes, LPS aggregate size distributions, and peptide-induced leakage kinetics of DOPE/DOPG liposomes, together with liposome size distributions in the absence and presence of KYE21 and WWWKYE21.
